# High Performance 3D PET Reconstruction Using Spherical Basis Functions on a Polar Grid

**DOI:** 10.1155/2012/452910

**Published:** 2012-04-02

**Authors:** J. Cabello, J. E. Gillam, M. Rafecas

**Affiliations:** ^1^Instituto de Física Corpuscular, Universitat de València/CSIC, Edificio Institutos de Investigación, 22085 Valencia, Spain; ^2^Departamento de Física Atómica, Molecular y Nuclear, Universitat de València, 46100 Valencia, Spain

## Abstract

Statistical iterative methods are a widely used method of image reconstruction in emission tomography. Traditionally, the image space is modelled as a combination of cubic voxels as a matter of simplicity. After reconstruction, images are routinely filtered to reduce statistical noise at the cost of spatial resolution degradation. An alternative to produce lower noise during reconstruction is to model the image space with spherical basis functions. These basis functions overlap in space producing a significantly large number of non-zero elements in the system response matrix (SRM) to store, which additionally leads to long reconstruction times. These two problems are partly overcome by exploiting spherical symmetries, although computation time is still slower compared to non-overlapping basis functions. In this work, we have implemented the reconstruction algorithm using Graphical Processing Unit (GPU) technology for speed and a precomputed Monte-Carlo-calculated SRM for accuracy. The reconstruction time achieved using spherical basis functions on a GPU was 4.3 times faster than the Central Processing Unit (CPU) and 2.5 times faster than a CPU-multi-core parallel implementation using eight cores. Overwriting hazards are minimized by combining a random line of response ordering and constrained atomic writing. Small differences in image quality were observed between implementations.

## 1. Introduction

Iterative statistical methods are the preferred reconstruction algorithms in emission tomography. Image quality greatly depends on how accurately physical phenomena are modelled in the system, which is represented as the system response matrix (SRM) [1, 2]. The SRM models the probability of detection, of an annihilation produced in voxel *b*, in a detector element or crystal pair *d*, *p*_*d*,*b*_. The size of the SRM is imposed by the number of voxels *N* that comprises the field of view (FOV) and by the number of detector elements of the scanner, *J*. The size of the SRM, *N* × *J*, is significant due to the large number of crystals used by high resolution scanners and the fine granularity of the FOV [3]. Fortunately, most elements *p*_*d*,*b*_ in an SRM are typically zero, which means that the number of nonzero SRM elements to store is significantly lower than *N* × *J*.

The SRM can be directly obtained by taking measurements where a point source is placed in different positions over the FOV, interpolating intermediate positions when needed. This produces a highly accurate SRM at the cost of long acquisition times [4, 5]. To greatly reduce acquisition time, a single scan of an array of point sources can be simultaneously acquired [6]. However, rather than directly measuring the system matrix such methods are used only to estimate the shift-variant point spread function. Alternatively, the SRM can be calculated using analytical methods. The speed of these methods outperforms others, at the expense of limited precision [7, 8]. Finally, the SRM can be calculated using Monte Carlo methods [9–11]. These methods produce a more accurate SRM than analytical methods, but do not include the physical phenomena that are only found by measuring the SRM. Regarding the time necessary to calculate the SRM, a simulation is longer than analytical computations, but less time consuming than that necessary to obtain measurements experimentally.

The high granularity of current PET scanners, used to obtain the best possible spatial resolution, makes the calculation of the SRM elements a cumbersome process. The use of cylindrical symmetries, taking advantage of the polygonal architecture of PET scanners, is a common approach to reduce the number of SRM elements required [9–15]. In the specific case of a Monte Carlo-based SRM, the simulation time and storage required can be greatly reduced.

Although the most common model used to represent the image space is the cubic voxel, there exists a number of alternative basis functions. The use of polar voxels provides a convenient model to exploit scanner symmetries, which significantly reduces the simulation time and space necessary to store the SRM only modelling a small portion of the FOV [16]. Alternatively, spherical basis functions (blobs) have been shown to represent a more suitable basis to model the continuous radiotracer distribution [17]. Improved noise performance has been demonstrated, compared to cubic and polar voxels [18–20]. Moreover, better spatial resolution can be obtained with spherical basis functions, compared to postreconstruction filtered cubic and polar voxels [15].

Spherical basis functions overlap in space. Therefore, the number of basis functions intersected by any given line of response (LOR) is higher using blobs compared to voxels. This characteristic produces that the SRM has a significantly large number of nonzero SRM elements, resulting in long CPU-based reconstruction times.

The forward and backward projections in a Maximum-Likelihood- (ML-) based reconstruction algorithm perform independent operations on each LOR and voxel, respectively, which makes these operations highly parallelizable. Graphical processing units (GPUs) technology have been successfully employed to enhance speed of image reconstruction in both precomputed [21–23] and on-the-fly [24, 25] SRMs, which makes it a potential candidate for blob-based reconstruction as well.

An alternative approach to parallelize the reconstruction process is to use CPU-multicore architectures. In this work we have implemented an optimized CPU-multicore version of the reconstruction algorithm using the Message-Passing-Interface (MPI) libraries (http://www.mcs.anl.gov/research/projects/mpi/index.htm).

This paper explores the suitability of these two approaches for the special case of ML-Expectation-Maximization (EM) PET reconstruction using a blob-based Monte Carlo precomputed SRM. The use of a large precomputed SRM in GPU technology represents one of the major challenges of this work.

## 2. Materials and Methods

### 2.1. Scanner Description

**Figure 1:**
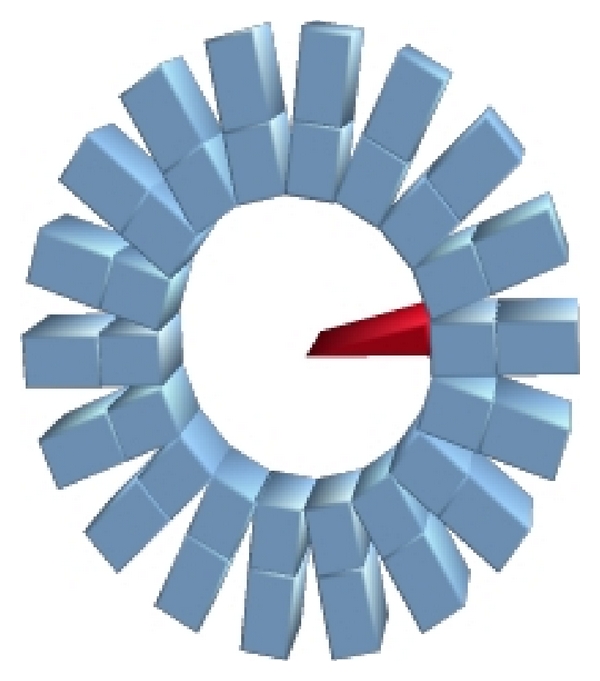
Mockup of MADPET-II with the wedge-like source simulated in red to calculate the SRM.

In this study the small animal scanner MADPET-II [26], shown in Figure 1, is used as a model for all the simulations carried out. MADPET-II has a radial diameter of 71 mm and an axial FOV of 18.1 mm. The ring contains 18 modules, where each module has two layers of 8 × 4 LYSO crystals with individually read out electronics based on avalanche photodiodes. The size of the crystals in the front layer is 2 × 2 × 6 mm^3^ and that of the rear layer is 2 × 2 × 8 mm^3^. The dual layer provides information of the depth of interaction, which mitigates parallax error.

### 2.2. Hardware Description

The graphics card used in image reconstruction was the NVIDIA Tesla C2070, based on a Fermi architecture. The GPU contains 448 cores (thread processors or shaders) running at 1.15 GHz (575 MHz core), 6 Gb GDDR5 on-board global memory, 48 Kb of shared memory per block, and a bandwidth of 144 Gb/s. Threads are internally organized as a grid of thread blocks, where threads in the same block communicate through shared memory. The maximum number of blocks is 65 535 and the maximum number of threads per block is 1 024. The card has a peak performance of 515.2 Gflops/s for double precision operations.

The algorithm was implemented for both the GPU and an eight-core desktop PC (Intel(R) Core(TM) i7 CPU 950 @ 3.07 GHz) with 12 Gb of RAM. An optimized algorithm allowed the use of either a single core or multiple cores and distributed memory.

### 2.3. Polar Symmetries and SRM Calculation

The total number of crystals of MADPET-II is 1 152; for image reconstruction using cubic voxels an image space of 140 × 140 × 40 (voxels of size 0.5 × 0.5 × 0.5 mm^3^) was used, resulting in *J* × *N* = 10^12^ elements. However, the SRM is highly sparse, significantly reducing the number of elements necessary for storage.

A popular method to reduce the number of SRM elements to calculate and to reduce the SRM storage size is to exploit the scanner symmetries. The typical polygonal architecture in PET scanners allows the use of polar symmetries. For a Monte Carlo-based SRM this approach allows not only the reduction of the file size of the SRM, but also the simulation time. The level of reduction depends on the number of symmetries which can be exploited. The number of symmetries in a PET system is intrinsically linked to its geometry, specifically to the number of detector modules employed over the circumference. The high resolution small animal scanner MADPET-II contains 18 block detectors. Using 18 rotational symmetries and 2 reflection symmetries transaxially and 2 reflection symmetries axially, up to 72 symmetries can be exploited.

 Therefore, a factor of 72 reduction in the simulation time and in the SRM file size is achieved. More details about this implementation of symmetries in MADPET-II can be found in [16].

The SRM was calculated using GATE [27] in the local GRID facility which had 108 nodes (two Quad Core Xeon E5420 @ 2.50 Ghz machines) for sequential processing. The total number of simulated events in the SRM was 3.7 × 10^10^, which represents 4.3 × 10^6^ events per voxel. The simulation of the 3.7 × 10^10^ events was split in 200 parallel jobs, where each parallel job took approximately 12 hours. The SRM was simulated using a back-to-back gamma source, thus half life, noncollinearity and positron range were ignored. A low energy threshold of 200 keV was applied at singles level. Singles list mode data were processed after simulation to select coincidences using a coincidence timing window of 20 ns. Accidental and multiple-scattered events are discarded in a postprocessing step.

### 2.4. Object Representation: Spherical Basis Functions

An estimation of the continuous radiotracer distribution *f*(*r*) is represented in image space as a linear combination of image coefficients and basis functions, expressed as

(1) $$\begin{array}{c} f\left(r\right)≃\hat{f}\left(r\right)=\overset{N}{\underset{i=1}{\sum }}c_{i}\psi \left(r-r_{i}\right), \end{array}$$

where *r* represents the space coordinate, $\hat{f}(r)$ the estimated distribution, *c*_*i*_ the image coefficients, *ψ*(*r*) the basis functions, *N* the total number of voxels, and *r*_*i*_ the placement grid.

Spherical basis functions provide better noise properties compared to cubic voxels. Spherical basis functions have compact support, that is, the function is zero beyond a given value (blob radius), but have a smoother behaviour than the traditional cubic voxels. The spherical basis functions used in this work are based on the Kaisser-Bessel function, described as

(2) $$\begin{array}{c} \psi {\left(r\right)}_{m,a,\alpha }=\frac{1}{I_{m}\left(\alpha \right)}{\left[\sqrt{1-{\left(\frac{r}{a}\right)}^{2}}\right]}^{m}I_{m}\left(\alpha \sqrt{1-{\left(\frac{r}{a}\right)}^{2}}\right), \end{array}$$

where *m* is the order of the Bessel function *I*, *a* is the blob radius, and *α* is the taper parameter. A thorough investigation of these parameters is found in [28] in which the optimal values are obtained. The study, performed in frequency space, determines that the optimal value of *m* is 2 (the first derivative is continuous at the blob boundary), the optimal radius *a* is 1.994Δ (Δ being the distance between elements of the underlying grid), and the optimal *α* is 10.4.

**Figure 2:**
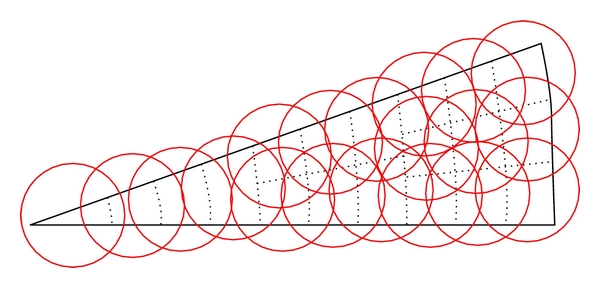
Wedge-like source simulated to calculate the SRM with blobs and underlying voxelized grid (not shown to scale).

Figure 2 shows the wedge-like source simulated to calculate the SRM elements using symmetries, with an underlying polar grid and blobs placed over the grid using a body-centred strategy with the polar voxels used as reference.

Using spherically symmetric basis functions and exploiting spherical symmetries, the final SRM was 5.3 Gb in size and contained 7.04 × 10^8^ nonzero elements (out of the 5.79 × 10^9^: 8740 blobs ×  662 976 LORs). If eight cubic symmetries were used (2 rotational + 2 reflectional transaxially + 2 reflectional axially), instead of spherical symmetries, the resulting SRM file size would be 47.2 Gb with the same statistical quality as that used.

### 2.5. Description of Phantoms

Three different phantoms were simulated for this study. All phantoms were simulated using a back-to-back gamma source in agreement with the simulation of the SRM. Therefore, only true coincidence events remained for image reconstruction. Simulated data is stored in LOR-histograms where each histogram bin corresponds to an LOR, and no preprocessing was applied to the data [9, 29]. The three phantoms used here are detailed below.

1. A homogeneously filled phantom with a hot and a cold rod inserts has been simulated to study the image quality. The cylinder is 20 mm long and 30 mm radius, while the rod inserts are 20 mm long and 10 mm radius. The simulated activity of the phantom was 32 MBq (0.86 mCi) and 74 MBq (2 mCi) in the hot and warm regions, respectively (3 : 1 ratio). The experiment time simulated was 155 seconds, producing a total of *∼*4.5 ×10^7^ coincidences.
2. To study the spatial resolution, an ellipsoidal phantom with six hot point sources and six cold spheres (1.5 mm *∅*), placed radially along the ellipsoid and separated by 5 mm on a warm background, was simulated. The ellipsoid was 35 mm by 20 mm transaxially and 2 mm long. The phantom was placed 12.5 mm off-centre covering more than one half of the FOV. The activity simulated in the point sources was 3.7 MBq (0.1 mCi) and 207.2 MBq (5.6 mCi) in the background (200 : 1 ratio), producing a total of *∼*9.6 ×10^6^ coincidences. The background was used in order to mitigate the resolution enhancement caused by the nonnegativity constraint of ML-EM [5, 30].
3. The digital mouse phantom MOBY [31] was simulated with a total activity of 0.2 mCi. Five bed positions were necessary to acquire the whole mouse with six overlapping slices (3 mm) between bed positions, with a time scan of four minutes per bed position. The activity simulated in each organ is the default relative activity set by the MOBY phantom files. It was not a purpose of this study to simulate realistic activity concentrations in each organ.

### 2.6. Quality Assessment: Figures of Merit

The noise performance obtained with each of the implementations explored was measured in the image quality phantom using three figures of merit, the coefficient of variation (CV), the contrast to noise ratio (CNR), and the correlation coefficient (CC). The spatial resolution was also studied by measuring the full width at half maximum (FWHM) across the point sources of the ellipsoidal phantom described in Section 2.5. These figures of merit were measured to compare the different implementations presented here, and not to assess the performance of spherical basis functions.

The CV and CNR were measured over regions of interest (ROI) of size 8 × 9 × 0.5 mm^3^, placed far enough from the boundaries so that there were no edge effects. The CC was measured over the entire image volumes. In all cases only one realization of the phantom was used.

The CV is commonly used as a normalized measurement of noise in a given ROI and is described as

(3) $$\begin{array}{c} \text{CV}=\frac{\sigma_{s}}{\mu_{s}}, \end{array}$$

where *μ*_*s*_ is the mean value and *σ*_*s*_ is the standard deviation measured in the ROI.

The CNR is a measure of noise performance between two ROIs [32] given by

(4) $$\begin{array}{c} \text{CNR}=\frac{\mu_{s}-\mu_{b}}{\sqrt{\left(\sigma_{s}^{2}+\sigma_{b}^{2}\right)/2}}, \end{array}$$

where *μ*_*b*_ is the mean value and *σ*_*b*_ is the standard deviation measured in the background.

The CC between two images is a statistical similarity measure defined as

(5) $$\begin{array}{c} {\text{CC}}_{AB}=\frac{\sum_{i=1}^{N}\left({\hat{f}}_{A_{i}}-{\bar{f}}_{A}\right)\left({\hat{f}}_{B_{i}}-{\bar{f}}_{B}\right)}{\sqrt{\left(\sum_{i=1}^{N}{\left({\hat{f}}_{A_{i}}-{\bar{f}}_{A}\right)}^{2}\right)\left(\sum_{i=1}^{N}{\left({\hat{f}}_{B_{i}}-{\bar{f}}_{B}\right)}^{2}\right)}}, \end{array}$$

where ${\hat{f}}_{A_{i}}$ is the intensity value at voxel *i* in image *A*, ${\bar{f}}_{A}$ is the mean value of image *A*, and similarly for image *B*. A CC value of 1 represents two perfectly correlated images, while a value of −1 represents two completely uncorrelated images.

The spatial resolution was measured as the FWHM taken from a profile drawn across the hot point sources embedded in the ellipsoidal phantom.

## 3. Hardware Implementations

### 3.1. GPU Implementation

The SRM was precomputed and stored in sparse format. One of the main challenges addressed in this work was the difficulty to cope with the amount of information needed by a single thread to perform both forward and backward projection, given that the SRM was too large to be stored in local memory, registers or shared memory. While a memory access to a register or shared memory takes only one clock cycle, access to global memory takes 400–600 clock cycles and access to texture memory takes 1–100 clock cycles (depending on cache locality).

The floating point values used in this work were distributed as follows: given the significant size of the SRM (5.3 Gb) this was stored in global memory, while the arrays stored in texture memory were the ratio between measurements and projected image estimate (2.52 Mb), a look-up table used to unfold the symmetries (2.40 Mb) and the image estimate used in the forward projection (2.40 Mb).

The number of blocks in the GPU grid was optimized to achieve the shortest reconstruction time per iteration, being 32 blocks obtained empirically.

The SRM was ordered by consecutive LOR indices in sparse format. Therefore, memory access to the SRM elements in the forward projection operation was consecutive, as opposed to the backward projection, where access to the SRM elements was highly irregular. The SRM organization implies that the forward projection was a gathering operation, while the backward projection was a scattering operation, which is slower.

During the forward projection, each thread reads from a number of blob coordinates and calculates the projection of one LOR, hence writing in different memory positions. However, during the backward projection, each thread reads from a number of measurements, back-projects the current estimate to image space, and writes in the corresponding blob coordinate. Given that threads are organized by LORs, different threads can write in the same memory position. This represents what is known as a race condition. To avoid such problem three different approaches have been implemented as follows.

(1) An SRM ordered by consecutive LOR indices (detection) was used in the forward projection, while an SRM ordered by consecutive blob indices (image) was used in the backward projection. This second SRM represents the transpose of the initial SRM although stored in a different file due to the use of sparse format. Using this approach the LOR-ordered SRM is loaded for the forward projection and subsequently unloaded. The blobs-ordered SRM is then loaded for the backward projection. The process was repeated for each iteration. The advantages are that the access to SRM elements was in consecutive memory positions for both operations and that there was no overwrite hazards. The disadvantage of this implementation is the time required for each iteration to load and unload each SRM file in the GPU. To refer to this implementation the term SRM_reload_ was used.
(2) An SRM ordered by consecutive LOR indices was used in both, the forward and backward projections. This approach represented an overwrite hazard during the backward projection when LORs intersecting the same voxels were processed by threads in parallel. This situation is likely to happen in those areas where most LORs intersect, that is, the centre of the FOV. Atomic writing prevents this situation from occurring, at the cost of longer computation time. To refer to this implementation the term SRM_atomic_ was used.
(3) Similarly to the implementation above, an SRM ordered by consecutive LOR indices was used in both, the forward and backward projections. To mitigate the speed problem caused by atomic operations, a combination of two strategies was followed:
  (a) the LORs were sent to the threads using a random ordering, hence not following any spatial correlation. By introducing spatial randomness in the execution of LORs in parallel, the probability of processing intersecting LORs simultaneously decreased drastically. However, writing in the same memory positions still happened in a region at the centre of the FOV;
  (b) atomic writing was exclusively used for those voxels located in the two central slices. Otherwise a nonatomic operation was performed.

To refer to this implementation the term SRM_rand-at_ was used.

### 3.2. Implementation Using the MPI Libraries

This approach used distributed memory, hence a portion of the SRM is sent to each core to perform the forward and backward projections, respectively. The SRM ordered by LOR indices was used in the forward projection, while a blobs-ordered SRM is used for the backward projection, similar to the GPU implementation using SRM_reload_. Subsequently, the SRM was loaded and unloaded each time a forward and a backward operation was performed, adding a computational overhead.

## 4. Results and Discussion

### 4.1. Timing Performance

The reconstruction time obtained in this work using GPU technology and spherical basis functions is comparable to the reconstruction time obtained using polar or cubic basis functions on a CPU [33]. However, the time performance is not as high as that published in other works [22, 25] due to the nature of this approach, that is, the use of a large Monte Carlo precomputed SRM. This implies that the number of global memory accesses by each thread in a forward/backward projection corresponds to the number of nonzero elements of each LOR/blob multiplied by the number of symmetries. This clearly represents the main bottle-neck in this approach.

**Figure 3:**
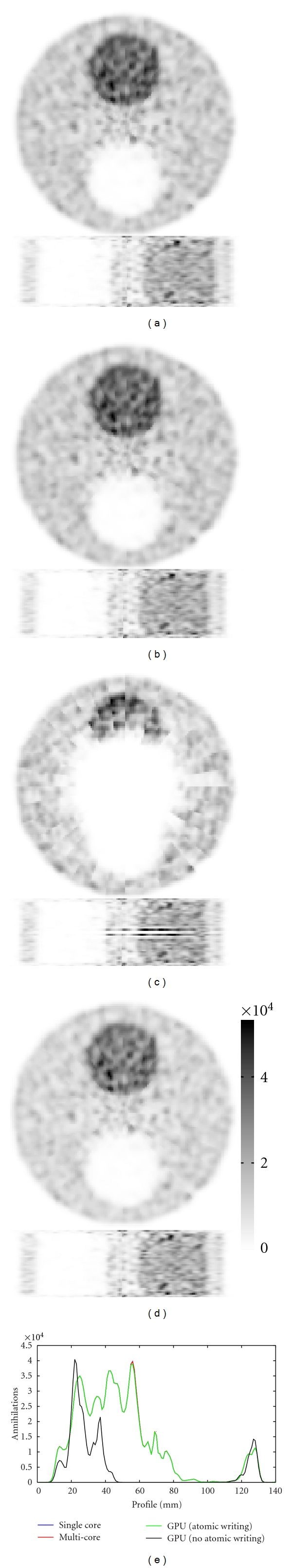
Transaxial and axial slices of the image quality phantom reconstructed after 300 iterations using a single-core CPU (a), eight cores parallelized using MPI libraries (b), the GPU with no atomic operation (c), and the GPU with atomic operation (d).

Table 1 shows a comparison between the time performance measured using a single Intel(R) Core(TM) i7 CPU 950 @ 3.07 GHz, that is, nonparallelized (np), with the time performance measured using the CPU-multicore implementation for 1 (MPI-1), 2 (MPI-2), 4 (MPI-4), and 8 (MPI-8) cores, and finally the time performance measured with the GPU, using the different implementations described above (SRM ignoring the overwrite hazard, SRM_reload_, SRM_atomic_, and SRM_rand-at_). The results shown in Table 1 were obtained using the image quality phantom described in Section 2.5. Nevertheless, small variations were observed between different phantoms. These reconstruction times represent average times measured after several iterations. The CPU-single-core implementation has been taken as reference to calculate the improvement factors of the parallelized implementations. Table 1 shows that the GPU-based implementation where the overwrite hazard is ignored is the fastest implementation because atomic writing is not used, at the cost of producing unacceptable artefacts in the final reconstructed images (see Figure 3(c)).

Using SRM_reload_, memory access to SRM elements is performed consecutively, both in the forward and backward projection. In every other implementation, consecutive memory access is performed only in the forward projection but not in the backward projection, hence decreasing the time performance. Using the implementation with SRM_reload_ the forward projection takes 97 s and 58 s to load the SRM, while the backward projection requires only 87 s and 57 s for loading. This represents an iteration time of 184 s if we consider only processing time. However, an extra 115 s is taken to load the SRM in each operation. Strict reconstruction time was very consistent for all the iterations. However, SRM loading/unloading time varied slightly for each iteration.

The atomic operation clearly increases the backward projection time. However, by using the atomic operation only for those critical voxels where the probability of over-writing is high, the backward projection time of implementation using SRM_rand-at_ is reduced to 132 s, close to the backward projection time measured in the implementation where the overwrite hazard is ignored (121 s). Moreover, the artefacts obtained in the reconstructed images when the overwrite hazard is ignored are removed using SRM_rand-at_ (Figure 3(d)).

### 4.2. Quantitative Assessment and Image Quality

When enhancing speed of an image reconstruction algorithm, it is of critical importance to produce the same image for each implementation. To demonstrate that the implementations detailed in this work do not have an impact on image quality, the phantoms described in Section 2.5 have been reconstructed using the CPU-single-core implementation, the CPU-multicore implementation using eight cores, and the GPU-based implementation using SRM_rand-at_ listed in Table 1.

#### 4.2.1. Noise Assessment

The impact on the noise performance has been assessed using the image quality phantom described in Section 2.5, which has been reconstructed after 300 iterations using four of the implementations studied (Figure 3): the CPU-single-core, the CPU-multicore, and two of the GPU-based implementations, without atomic operation and with atomic operation used only in the two central slices (SRM_rand-at_). Special mention is required for the GPU-based implementation without atomic writing (Figure 3(c)) where significant artefacts are observed, mainly in the centre of the FOV. As explained above, these artefacts are due to parallel threads overwriting in the same memory positions during the backward projection, due to the high overlapping between LORs in the centre of the FOV. The artefacts are removed by performing an atomic operation (Figure 3(d)).

The images obtained with SRM_reload_ and SRM_atomic_ (not shown in this work) are practically identical to the one obtained with SRM_rand-at_. A profile across the four reconstructed phantoms is shown in Figure 3(e), demonstrating great resemblance between the images obtained with the CPU-single-core, the CPU-multicore, and the GPU-based implementation with atomic writing, while the artefact observed in Figure 3(c) is clearly visible in Figure 3(e) in the black profile.

**Figure 4:**
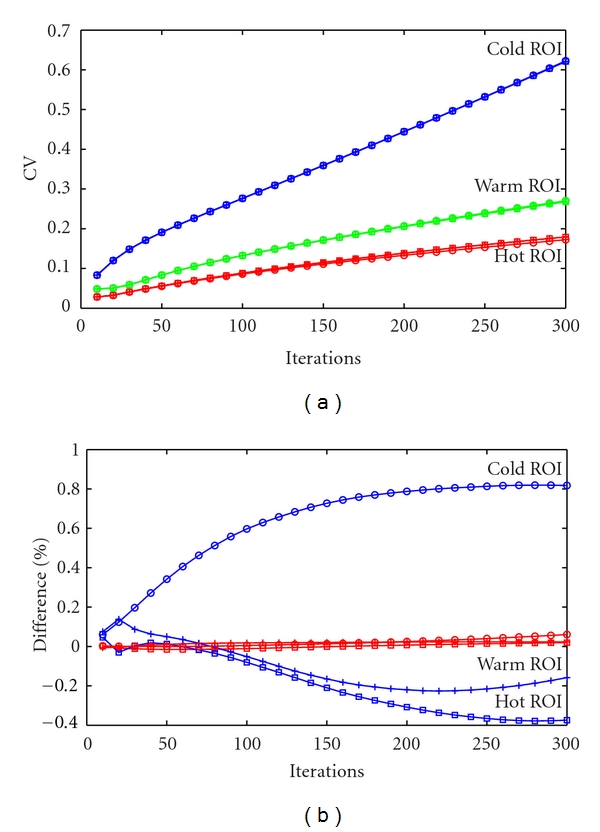
CV measured in the hot (red), warm (green), and cold (blue) ROI for the CPU-single-core implementation (□), the CPU-multicore implementation (+), and the GPU implementation (∘) for 300 iterations measured every 10 iterations (a). The differences in % between the CPU-single-core/CPU-multicore (red) and CPU-single-core/GPU (blue) implementations for the hot (□), warm (+), and cold (∘) ROI are shown in (b).

For quantitative assessment, the CV (Figure 4) was measured in the hot, warm, and cold ROIs for the three different implementations, every 10 iterations for 300 iterations. The CV at iteration 300 is 0.15 and 0.28 for the hot and warm ROIs, respectively, for all three implementations, while the CV in the cold ROI for the CPU-based implementations is 0.72 and for the GPU-based implementation 0.73. In all cases the CV follows an increasing trend due to the known noise increase as more ML-EM iterations are calculated. Differences between the CPU-based implementations (single-core and multicore) are below 0.08% (Figure 4(b)), while higher differences were observed between the CPU-single-core and GPU-based implementations, where differences between −0.37% (hot ROI) and 0.81% (cold ROI) were measured. These differences are due to the different floating point precisions available in the CPU and the GPU. While the precision had little effect on individual SRM elements, the cumulative effect produced differences in the reconstructed images. However, these differences are expected to be dominated by statistical errors in the data.

**Figure 5:**
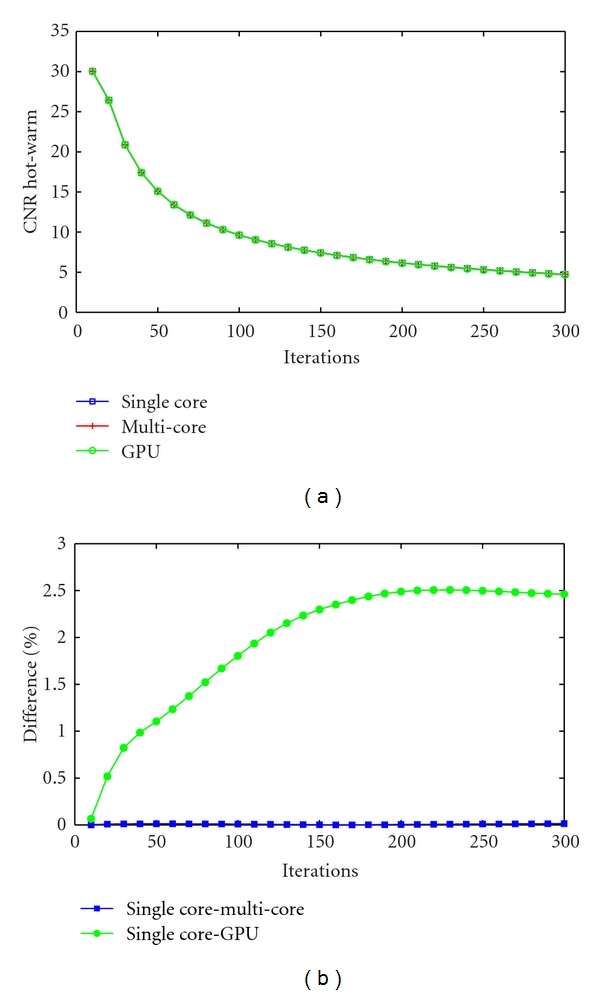
CNR between the hot and warm ROIs for 300 iterations measured every 10 iterations (a) and CNR difference in % between the CPU-single-core/CPU-multicore and CPU-single-core/GPU implementations (b).

Figure 5 shows the evolution of the CNR measured between the hot and warm ROIs for 300 iterations at every 10 iteration. The differences between the CPU-based and the GPU-based implementations are shown in Figure 5(b). Similarly to the CV study, differences between the CPU-based implementations (single-core and multicore) are below 0.02%, while small differences are observed between the CPU-single-core and the GPU implementations. The maximum difference is 2.5% and stabilizes after 200 iterations. However, from Figure 3 the images are visually indistinguishable.

**Figure 6:**
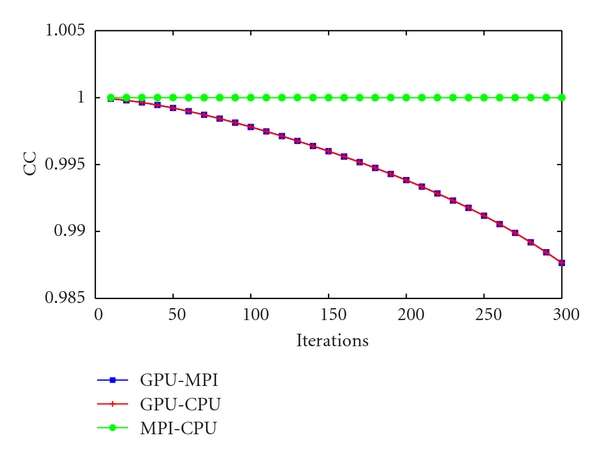
CC measured between the CPU-multicore/CPU-single-core, GPU/CPU-single-core, and GPU/CPU-multicore implementations for 300 iterations measured every 10 iterations.

Finally, the CC (Figure 6) was measured between the entire volumes of the resulting reconstructed image quality phantoms obtained with the CPU-based implementation, the MPI-based implementation, and the GPU-based implementation. Similarly to the CV and the CNR studies, the CC measured between the CPU-based and the MPI-based implementations shows perfect correlation, while the comparison between the GPU-based implementation with the CPU-based and the MPI-based implementations show high correlation initially, but the trend is to slightly decrease at later iterations. However, the CC at 300 iterations is over 98.5% so that the difference can be considered negligible. To confirm that the trend was not exacerbated as more iterations are computed, the CC between the MPI-based implementation and the GPU-based implementation was computed over 600 iterations, showing a CC of 94% at 600 iterations and a slight trend correction.

#### 4.2.2. Spatial Resolution Assessment

**Figure 7:**
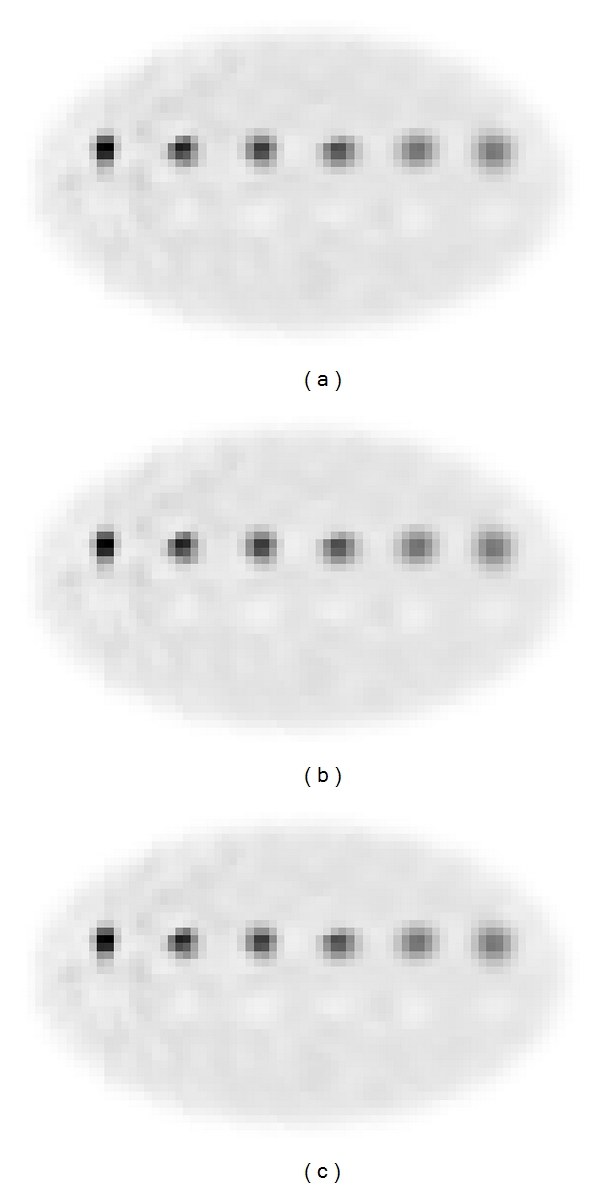
Ellipsoidal phantom with embedded point sources reconstructed after 300 iterations using a CPU-single-core (a), eight cores parallelized using MPI libraries (b), and the Tesla GPU (c).

Figure 7 shows the spatial resolution phantom reconstructed after 300 iterations using the CPU-single-core, the CPU-multicore, and the GPU-based implementations (using SRM_rand-at_). The three reconstructed phantoms show great resemblance by visual inspection.

**Figure 8:**
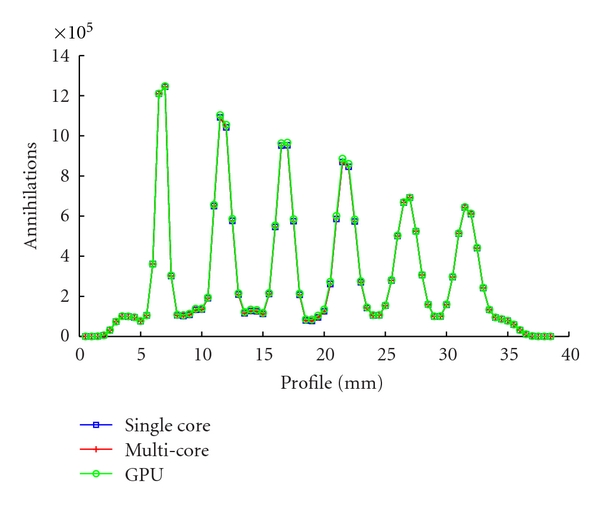
Profile across the point sources in phantom shown in Figure 7 reconstructed after 300 iterations using a CPU-single-core (a), eight cores parallelized using MPI libraries (b), and the Tesla GPU (c).

The profile drawn across the point sources in the three reconstructed phantoms, shown in Figure 8, confirms the conclusions made in the noise study. The CPU-single-core and the CPU-multicore implementations produce identical results while the images reconstructed using the GPU implementation are slightly different. This is further confirmed by Table 2, where the FWHM (mm) measured from each point source and each profile is shown (Ps 1 corresponds to the point source located closer to the centre of the FOV while Ps 6 corresponds to the point source located closer to the edge of the FOV). The spatial resolution decreases as the point source is located farther from the centre of the FOV due to the parallax effect.

**Figure 9:**
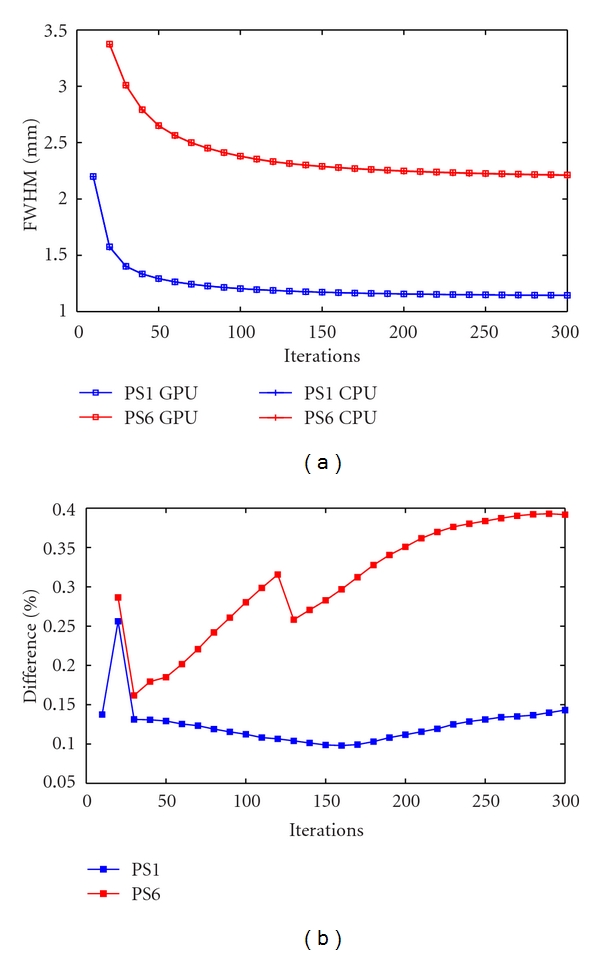
FWHM (mm) of the closest (1-blue) and farthest (6-red) point source from the centre of the FOV for 300 iterations measured every 10 iterations obtained with the CPU (+) and the GPU (□) (a) and difference in % between the CPU and GPU implementations for each point source (b).

Similarly to the noise assessment study, the FWHM was measured over 300 iterations at steps of 10 iterations (Figure 9(a)), showing small differences measured between the CPU-based and the GPU-based implementations below 0.4% at 300 iterations (Figure 9(b)).

### 4.3. Qualitative Assessment

For qualitative assessment, the MOBY phantom [31] has been reconstructed using blobs in the GPU and using the CPU with the MPI libraries. For comparison purposes, the phantom was also reconstructed using traditional cubic voxels. However, it is important to highlight that the focus of this work is not to compare these two basis functions. Spherical basis functions provide better noise performance than cubic voxels, so in order to perform a fair comparison, the image reconstructed using cubic voxels was filtered to match the noise performance of that achieved using spherical basis functions [33], which produces a visible image detail degradation.

**Figure 10:**
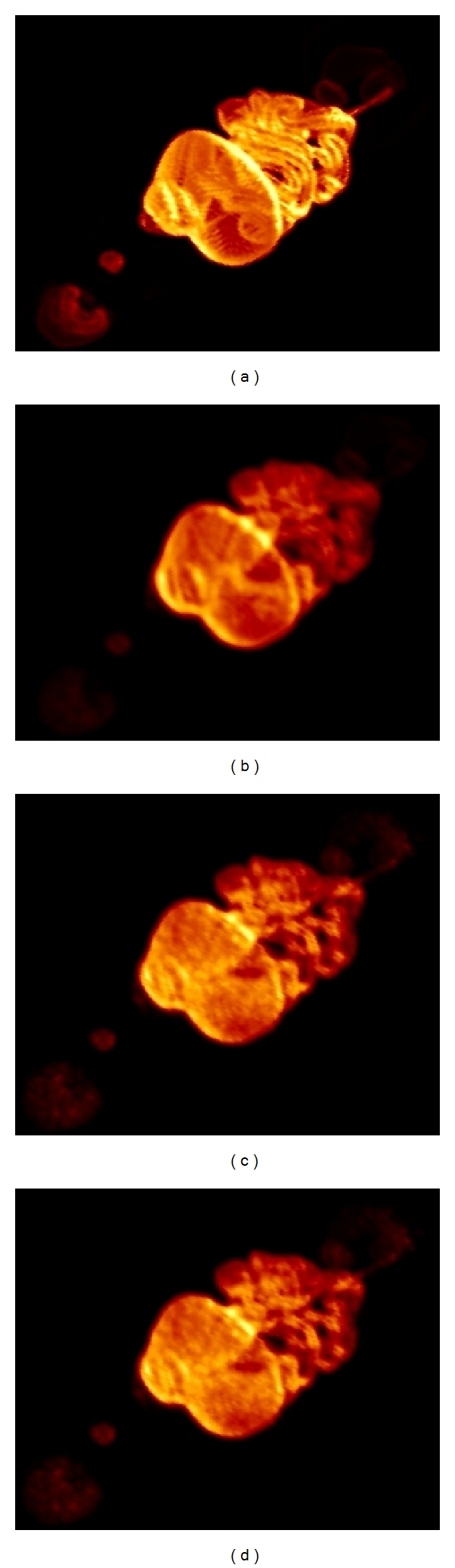
3D rendering of the ideal MOBY phantom (a), reconstructed phantom after 300 iterations using cubic basis functions filtered with a *σ* = 0.5 mm Gaussian filter (b), spherically symmetric basis functions using the GPU-based implementation (c), and CPU-multicore-based implementation (d).

Figure 10(a) shows the ideal MOBY phantom.  Figure 10(b) shows the reconstructed phantom obtained using cubic voxels with a postreconstruction Gaussian filter of *σ* = 0.5 mm for comparison purposes. A *σ* = 0.5 mm for the Gaussian filter has been applied to match the noise levels obtained with cubic voxels and blobs.  Figure 10(c) shows the phantom reconstructed with spherically symmetric basis functions using the GPU implementation and Figure 10(d) shows the phantom reconstructed with spherically symmetric basis functions using the CPU-multicore implementation. 300 iterations were used to reconstruct each bed position in the three reconstructed MOBY phantoms presented here.

It can be noticed that the thyroids and brain are more visible, and boundaries better delineated using spherical basis functions, compared to filtered cubic voxels (Figure 10(b)), as shown in Figures 10(c) and 10(d).

The reconstruction time necessary using cubic voxels in a single core in the CPU (Figure 10(b)) was *∼*110 hours, the CPU-multicore implementation using spherical basis functions (Figure 10(d)) was *∼*220 hours, and the GPU-based implementation using spherical basis functions (Figure 10(c)) was *∼*88 hours. If the same phantom was reconstructed using spherical basis functions on a single core, the necessary reconstruction time would be *∼*380 hours.

From Figure 10 it can be seen that the combination of blob-based reconstruction and a precomputed Monte Carlo SRM using GPU technology is a feasible alternative, not only for simple phantom geometries as those shown in Section 4.2, but also for multistage and highly realistic phantoms as the MOBY phantom.

## 5. Conclusions

Increasing granularity in PET scanners provides improved spatial resolution while increasing the number of detector elements at the same granularity improves sensitivity. However, increased resolution means that the calculation of the SRM is an extremely cumbersome task, particularly for simulation-based SRM calculation. Nevertheless, Monte Carlo-based system matrices for iterative statistical image reconstruction applied to emission tomography are growing in popularity due to their image quality advantages. The extended availability of affordable computing power means that significant efforts are being put into sophisticated improvements of the system response model.

Overlapping spherically symmetrical basis functions have clear advantages over nonoverlapping (cubic or polar voxels) even at the cost of a significantly high number of nonzero elements in the SRM, resulting in large SRM file sizes and long reconstruction times. These problems can be partly overcome by exploiting cylindrical symmetries to reduce the simulation time, the number of nonzero SRM elements, and hence the file size necessary to store the SRM. The combination of spherically symmetric basis functions and cylindrical symmetries makes this approach feasible for use in a clinical or preclinical application. However, reconstruction time is then the main concern due to the still large number of nonzero SRM elements required to process the forward and backward projection.

Ordered-Subsets- (OS-) EM represents a common approach to speed up the reconstruction process. While OS-EM can be implemented in GPU technology, it requires a device-dependent level of complexity, and its inclusion may reduce the generality of the study presented here. Moreover, subset choice interacts with both speed and image quality while hardware-based solutions decouple this relationship. While it is expected that the combination of OS-EM and GPU technology can effectively further reduce reconstruction times, this is beyond the scope of this investigation.

This work presents a SRM-generic hardware implementation that achieved reconstructed images 4.3 times faster using GPU technology compared to an optimized CPU-single-core implementation and 2.5 times faster than a CPU-multicore (8) implementation. A CPU-multicore implementation decreased the reconstruction time by only 1.7 times compared to the single-core implementation.

Differences in image performance were assessed from an image quality and a spatial resolution perspective. Negligible differences were demonstrated between CPU-based single-core and multicore implementations, and small differences between CPU-single-core and GPU-based implementations were observed. Differences below 1% for the CV, below 2.5% for the CNR, and below 0.4% for the spatial resolution were measured between the CPU-single-core implementation and the GPU-based implementation.

## Figures and Tables

**Table 1:** Time performance.

Platform	Forward	Backward	Iteration (s)	Improvement
	projection (s)	projection (s)		factor
CPU (np)	417	488	905	1

CPU (MPI-1)	523 (0.79)	748 (0.65)	1,272	0.71
CPU (MPI-2)	315 (1.32)	560 (0.87)	875	1.03
CPU (MPI-4)	214 (1.94)	357 (1.36)	571	1.58
CPU (MPI-8)	209 (1.99)	316 (1.54)	526	1.72

GPU (Overwrite hazard ignored)	79 (5.27)	121 (4.03)	200	4.52
GPU (SRM_reload_)	(58+)97 (2.7)	(57+)87 (3.38)	(115+)184	3.03
GPU (SRM_atomic_)	79 (5.27)	232 (2.10)	311	2.90
GPU (SRM_rand-at_)	79 (5.27)	132 (3.69)	211	4.28

**Table 2:** FWHM (mm) of the profiles shown in Figure 8.

Platform	Ps 1	Ps 2	Ps 3	Ps 4	Ps 5	Ps 6
CPU-single-core	1.17	1.65	1.73	1.96	2.25	2.24
CPU-multicore	1.17	1.65	1.73	1.96	2.25	2.24
GPU	1.17	1.66	1.74	1.96	2.26	2.24
